# Tezepelumab alters airway epithelial transcriptional responses and epithelial–smooth muscle signalling in uncontrolled asthma

**DOI:** 10.3389/fimmu.2026.1911911

**Published:** 2026-10-16

**Authors:** Sangeetha Ramu, Sara Vázquez-Mera, Mandy Menzel, Samuel Cerps, Juan José Nieto-Fontarigo, Morten Hvidtfeldt, Celeste Porsbjerg, Asger Sverrild, Lena Uller

**Affiliations:** 1 Unit of Respiratory Immunopharmacology, Department of Experimental Medical Science, Lund University, Lund, Sweden; 2 BioLympho Research Group, Department of Biochemistry and Molecular Biology, Faculty of Biology-Biological Research Centre (CIBUS), University of Santiago de Compostela, Santiago de Compostela, Spain; 3 Translational Research in Airway Diseases Group (TRIAD), Health Research Institute of Santiago de Compostela (IDIS), Santiago de Compostela, Spain; 4 Aquatic One Health Research Center (ARCUS), Universidade de Santiago de Compostela, Santiago de Compostela, Spain; 5 Department of Respiratory Medicine, University Hospital Bispebjerg, Copenhagen, Denmark

**Keywords:** alarmins, asthma, epithelium, smooth muscle cells, tezepelumab, transcriptomics

## Abstract

**Background:**

Tezepelumab is a monoclonal antibody that targets thymic stromal lymphopoietin (TSLP). It reduces airway hyperresponsiveness, eosinophilia, mucus plugging, and asthma exacerbations, which are commonly caused by viral infections. Although clinical TSLP blockade attenuates virus-induced IL-33 and T2 cytokines in human bronchial epithelial cells (HBECs), the underlying mechanisms remain incompletely understood.

**Aim:**

The aim of this study was to identify transcriptomic changes modulated by tezepelumab in HBECs and determine their downstream remodelling-related effects on bronchial smooth muscle cells (BSMCs).

**Methods:**

HBECs from patients with uncontrolled asthma before and after 12 weeks of tezepelumab (*n* = 13) or placebo (*n* = 13) were expanded *in vitro* as submerged monocultures and subsequently exposed to poly(I:C). Transcriptomic profiling was performed, and selected targets were validated at mRNA and/or protein levels. Conditioned media from HBECs were applied to BSMCs to assess the expression of remodelling-associated mediators.

**Results:**

Tezepelumab treatment downregulated S100A8 and S100A9 expression in unstimulated HBECs. Following poly(I:C) stimulation, exploratory transcriptomic analysis identified enrichment of TNF-α/nuclear factor kappa B (NF-κB)-related pathways in placebo-treated HBECs compared with tezepelumab-treated HBECs. Validation analyses showed variable responses across the cohort, with attenuation observed primarily in patients with higher baseline inflammatory responses. In separate gain-of-function experiments, recombinant TSLP enhanced poly(I:C)-induced expression of S100A8, S100A9, TNFSF15, and TRAF1, together with increased NF-κB activation. Furthermore, conditioned media from poly(I:C)-stimulated HBECs collected after tezepelumab treatment induced lower TGF-β2 gene expression in BSMCs than conditioned media from placebo-treated HBECs.

**Conclusion:**

Clinical TSLP blockade by tezepelumab was associated with altered epithelial transcriptional responses and reduced TGF-β2 gene expression in BSMCs exposed to epithelial conditioned media. These findings are hypothesis-generating and provide transcriptional insight into the mechanisms underlying the therapeutic benefits in asthma management.

## Graphical Abstract

**Figure:**
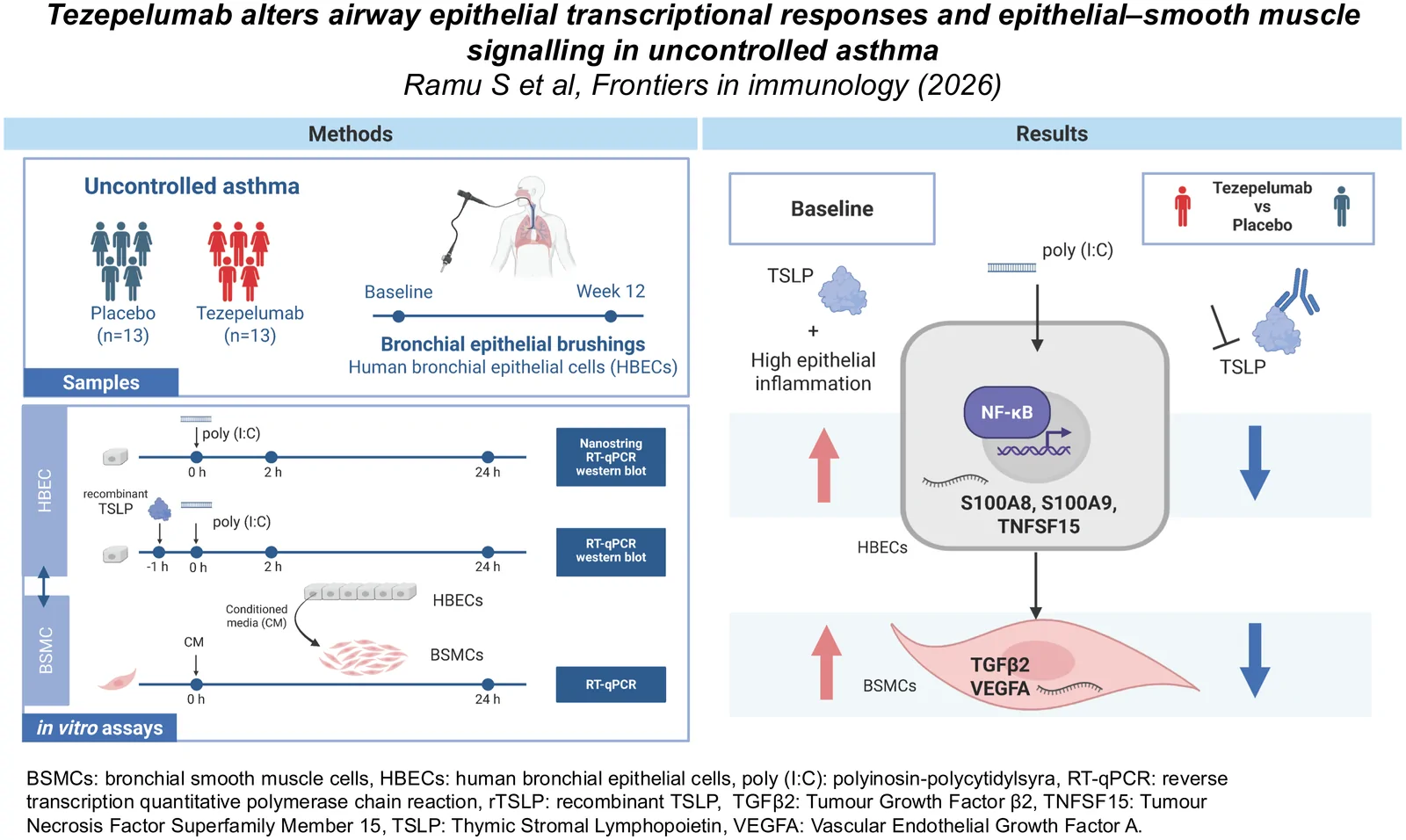
Created with BioRender.com.

## Introduction

The airway epithelium functions as a vital interface between the external environment and the respiratory system, serving as both a physical and an active immunological barrier that senses environmental triggers (1, 2). In this process, epithelial alarmins including interleukin (IL)-33, thymic stromal lymphopoietin (TSLP), IL-25, high mobility group box 1 (HMGB1), and calgranulins (S100 proteins) play central roles (3). Amongst them, the S100A8 and S100A9 proteins, which form a heterodimer known as calprotectin, activate key innate immune receptors such as Toll-like receptor 4 (TLR4) and the receptor for advanced glycation end products (RAGE), amplifying airway inflammation (4).

In asthma, particularly in severe disease, rhinoviral (RV) infection is a common cause of asthma exacerbation, characterised by an imbalanced release of cytokines and antiviral interferons in the airway epithelium (5, 6). This dysregulation may be due to genetic and/or epigenetic changes (7) that influence key molecular pathways, suggesting that the airway epithelium not only initiates immune responses but also sustains the proinflammatory environment driving airway remodelling.

Bronchial smooth muscle cells (BSMCs) undergo significant structural and functional remodelling in asthma (8), contributing to the development of airway hyperresponsiveness (AHR) (9). These processes are further enhanced by epithelial-derived cytokines and alarmins, including TSLP and IL-33 (10). We have previously shown that mediators released from human bronchial epithelial cells (HBECs) modulate the inflammatory responses of BSMCs, increasing IL-33 production (11). Additionally, BSMCs, together with epithelial cells, are now recognised as active contributors to immunomodulation and airway inflammation through expression of pattern recognition receptors (PRRs) and release of various cytokines and other mediators in response to external triggers (11, 12). Consistently, blockade of TSLP improves AHR in response to mannitol in patients with uncontrolled asthma (13), highlighting the therapeutic potential of targeting epithelial–mesenchymal crosstalk.

Tezepelumab, an anti-TSLP monoclonal antibody, reduces exacerbations in both T2-high and T2-low asthma patients (14). A mechanistic substudy of the randomised, double-blind, placebo-controlled phase 2 UPSTREAM trial revealed that tezepelumab significantly reduced IL-33 levels in bronchoalveolar lavage fluid (BALF) after 12 weeks in patients with uncontrolled asthma (15). Additionally, tezepelumab suppressed virus-induced IL-33 production in HBECs and decreased T2 cytokine expression, whilst preserving type I and III interferon responses (15). These findings suggest that targeting epithelial alarmins, particularly TSLP, may restore dysregulated epithelial immunity and exert broader anti-inflammatory effects through modulation of IL-33 and T2 cytokine pathways. However, the impact of tezepelumab on the epithelial transcriptome and downstream effects remains incompletely understood.

This study aimed to elucidate the molecular effects of tezepelumab treatment in the clinical setting on the *in vitro* virus-induced response of the bronchial epithelium and its impact on airway remodelling. We used a transcriptomic approach with a multiplex gene expression panel (NanoString) combined with *in vitro* validations in primary HBECs from patients with uncontrolled asthma isolated before and after treatment with tezepelumab or placebo. Functional assays were also performed to assess the effects of epithelial-derived mediators on BSMCs.

## Materials and methods

### Patients and ethics

HBECs were obtained from the UPSTREAM study and expanded as previously described (15). Samples were collected before and after treatment with tezepelumab or placebo. All participants provided informed consent, and the study was approved by the Local Ethics Committee in Copenhagen (H-16002008) and the Danish Medicines Agency (2016020256). Clinical characteristics have been reported previously (15).

### Bronchial epithelial cell cultures and *in vitro* stimulation

HBECs were obtained from patients before and after tezepelumab (*n* = 13) or placebo (*n* = 13) treatment and maintained as submerged monolayer cultures under standard conditions, as previously described (15). For each donor, a single culture was analysed for each experimental condition before and after treatment, resulting in 13 biological replicates per treatment group. All cell experiments were performed at passage 2, and cells were seeded into collagen-coated 12-well plates and grown to 70%–80% confluence in BEGM medium (Lonza, Walkersville, MD, USA). The growth medium was then replaced with bronchial epithelial basal medium (BEBM) supplemented with 1% insulin-transferrin-selenium (ITS) and 1.2% bovine serum albumin (BSA). HBECs were stimulated with the viral mimic poly(I:C) (10 μg/ml; Invitrogen Ltd, Paisley, UK) alone for 2 h and 24 h. For recombinant TSLP (rTSLP) experiments, HBECs were prestimulated with 2 ng/ml rTSLP (R&D Systems Inc., Minneapolis, USA) for 1 h and then harvested after 3 and 24 h of 10 μg/ml poly(I:C) (Invitrogen Ltd., Paisley, UK) stimulation. These experiments were performed using HBECs from six or seven independent donors before placebo/tezepelumab treatment (one independent experiment per donor). Cell lysates and supernatants were collected for further analysis described below.

### Bronchial smooth muscle cell culture and stimulation with conditioned media from HBECs

BSMCs from a healthy control obtained from Lonza (Walkersville, MD, USA) were cultured in T75 flasks under standard conditions (5% CO_2_ and at 37 °C) in SmBM^®^ Basal Medium supplemented with SmGM^®^-2 SingleQuots^®^ and 5% FBS (Lonza, Walkersville, MD, USA). Passages 6–7 were used in all experiments. BSMCs were seeded into 12-well plates (Nunc, Life Technologies, Carlsbad, CA, USA) for experiments.

BSMCs were treated with epithelial cell culture supernatants from unstimulated or poly(I:C) stimulated (24 h) HBEC-derived conditioned media (CM; diluted 1:10) (11) obtained from donors from the UPSTREAM (15) cohort who received 12 weeks of treatment with tezepelumab or placebo. All stimulations were performed in the above-mentioned bronchial smooth muscle growth medium with supplements (Lonza, Walkersville, MD, USA). Cell lysates and culture supernatants were collected after 24 h for protein and mRNA expression analyses as previously described (11).

### NanoString analysis

Isolated RNA from unstimulated and poly(I:C)-stimulated HBECs (15) was hybridised with the NanoString nCounter Immunology Human V2 panel code set (NanoString, Seattle, WA, USA). mRNA expression of 594 genes (including 15 housekeeping genes) was quantified using the nCounter Digital Analyzer at SCIBLU Genomics, LTH, Lund University, Sweden.

### Gene analysis by RT-qPCR

Total RNA was extracted from both HBECs and BSMCs using the RNeasy Plus Mini Kit (Qiagen, Stockholm, Sweden), and 0.5 to 1 µg of RNA was then reverse transcribed (High-Capacity cDNA Reverse Transcription Kit, Applied Biosystems, (Thermo Fisher Scientific, Waltham, MA, USA)) according to the manufacturer’s instructions. Gene expression was quantified using the following primers: Hs_S100A8_1_SG; Hs_S100A9_1_SG; Hs_VEGFA_6_SG; Hs_TGFB2_1_SG; Hs_TRAF1_va.1_SG; and Hs_TNFSF15_1_SG (Qiagen, Stockholm, Sweden) using an AriaMx real-time PCR system (Stratagene, La Jolla, CA, USA) with standard cycling parameters. The genes of interest were normalised to the geometric mean of two reference genes, ubiquitin c (UBC; Hs04931876_m1) and glyceraldehyde 3-phosphate dehydrogenase (GAPDH; Hs02786624_g1).

### Western blot analysis

Protein expression in cell lysates was quantified by Western blotting, as previously described (16). For that, we used the following primary rabbit antihuman antibodies from Cell Signaling (Danvers, MA, USA) at 1:1,000: phospho-nuclear factor kappa B (NF-κB) p65 (No. 3033), total-NF-κB p65 (No. 4764), IκBα (Inhibitor of nuclear factor kappa B alpha) (No. 9242S), β-tubulin (No. 2146S), and GAPDH (No. 5174S). Anti-rabbit secondary antibody conjugated to HRP (No. 09/2029; Cell Signaling, Danvers, MA, USA) was used at 1:5,000. Optical density was measured using a LI-COR Odyssey Fc imager system (LI-COR, Lincoln, NE, USA). To assess NF-κB pathway activation, phospho-NF-κB p65 signal was normalised to total NF-κB p65 to account for differences in total p65 protein expression. GAPDH was used as the loading control to verify even protein loading and transfer efficiency. Quantified protein levels were relativised as indicated in the corresponding figure legends. IκBα protein expression was normalised to β-tubulin. Data are presented as after/before treatment with tezepelumab or placebo (control_after_/control_before_ and poly(I:C)_after_/poly(I:C)_before_).

### ELISA analysis of S1008A/S1009A protein release in cell culture supernatants

S1008A/S1009A protein levels were measured in bronchial epithelial cell supernatants using a specific enzyme-linked immunosorbent assay (ELISA) with matched antibodies from R&D Systems (Minneapolis, MN, USA) according to the manufacturer’s instructions.

### Statistical analysis

For NanoString data, normalisation and differential gene expression analysis were performed using the DESeq2 package in R 4.4.3 (17). A generalised linear model was fitted including visit, treatment, and their interaction (visit × treatment). Reference levels were set to before treatment and placebo. For overrepresentation analysis (ORA) (*p*-value = 0.05), the Molecular Signatures Database (MSigDB) and the R package “clusterProfiler” were used. To identify genes included in specific Gene Ontology (GO) terms related to airway remodelling processes, the Genome-wide Annotation for Human database and the “org.Hs.eg.db” R package were employed.

Principal component analysis (PCA) and hierarchical clustering were performed in R (version 4.5.1) using the FactoMineR, factoextra, dplyr, and ComplexHeatmap packages. The included variables were baseline fractional exhaled nitric oxide (FeNO; ppb) levels and poly(I:C)-induced levels of the cytokines TNF-α, T2 cytokines (IL-4, IL-5, and IL-13), and the epithelial alarmin TSLP from HBECs collected before tezepelumab treatment. Data were scaled before being used as input. Hierarchical clustering was performed using the Euclidean distance and Ward’s minimum variance method (Ward.D2) to identify sample groupings. The resulting clusters were visualised as a heatmap, in which both variables and donors were ordered according to the hierarchical dendrogram. The average silhouette width (computed with the cluster package) was used to evaluate cluster robustness. Based on this clustering, tezepelumab-treated patients were stratified into distinct subgroups for subsequent analyses.

Data were analysed using GraphPad Prism version 10.0 (San Diego, CA, USA). Normality was assessed using the Shapiro–Wilk test. For tezepelumab vs. placebo (unpaired) comparisons (after/before treatment), ordinary one-way ANOVA with Šídák’s multiple comparisons test was used for normally distributed data, or Kruskal–Wallis test followed by Dunn’s multiple comparisons test for non-parametric data. For comparisons derived from rTSLP experiments, paired analyses were performed and corrected with the Geisser–Greenhouse correction. Given the small sample size (*n* ≤ 7) after patient stratification, group comparisons were performed using the nonparametric Mann–Whitney test, regardless of data distribution. Outliers (ROUT method, *Q* = 1%) were removed where indicated in the figure legends. *p*-values < 0.05 were considered statistically significant.

## Results

### S100A8 and S100A9 are downregulated in unstimulated HBECs following tezepelumab treatment compared with placebo

The effect of tezepelumab on the epithelial transcriptome in response to viral stimulation *in vitro* has not been studied. To investigate this, we conducted exploratory multiplex gene expression analysis (NanoString) on HBECs obtained from 11 asthma donors before and after placebo (*n* = 2) or tezepelumab (*n* = 9) treatment, at baseline and after poly(I:C) stimulation. After normalisation to placebo, only two out of 594 genes were found to be differentially expressed genes (DEGs) after tezepelumab treatment (FDR < 0.1) under unstimulated conditions: S100A8 and S100A9 (**Figures 1A, B**; **Supplementary Table S1**). A similar downregulation of these two genes was also observed in poly(I:C)-stimulated cells after tezepelumab treatment (**Supplementary Table S2**).

**Figure 1:**
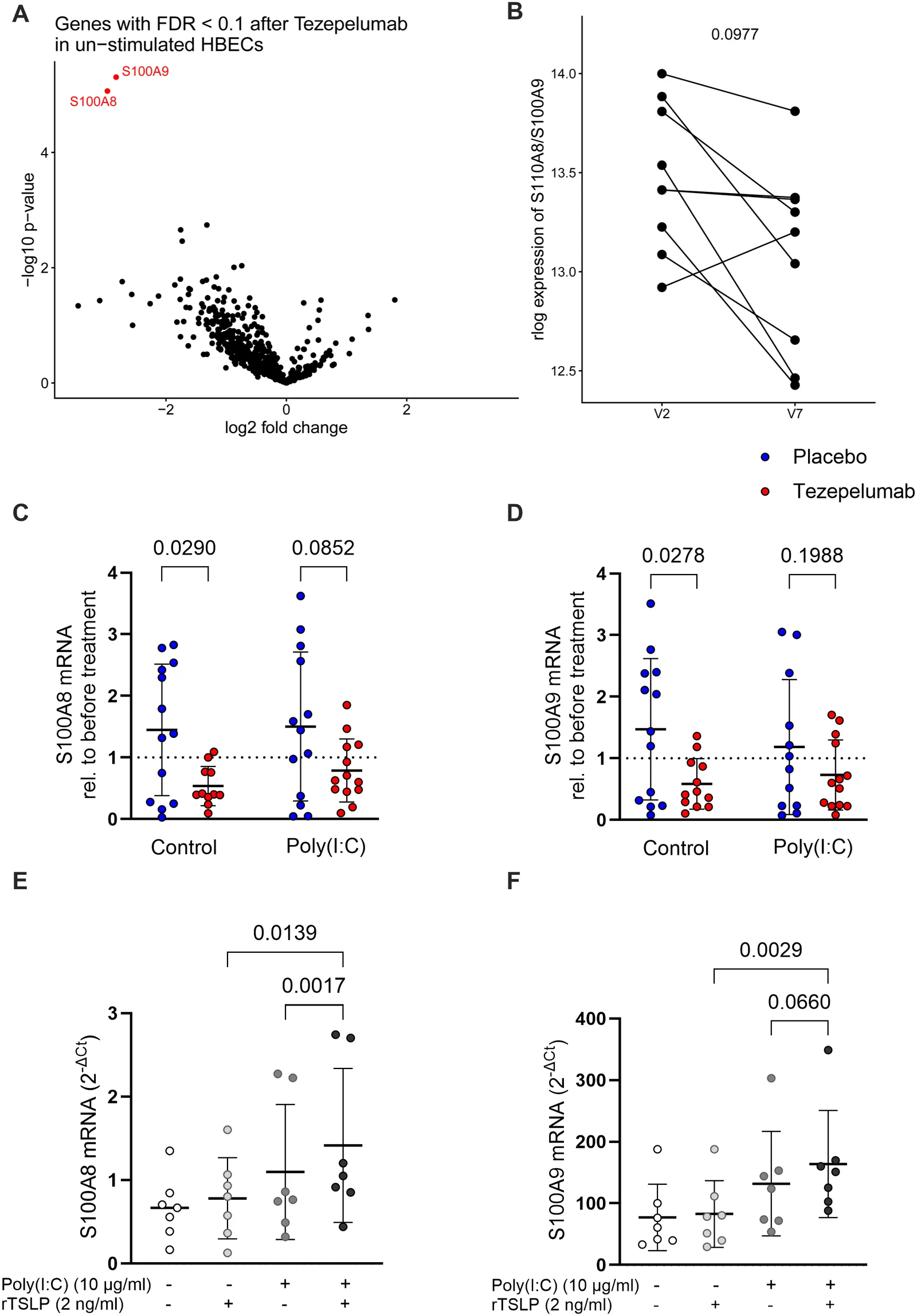
Effects of tezepelumab treatment and rTSLP on S100A8 and S100A9 gene expression in human bronchial epithelial cells (HBECs). **(A, B)** Differential gene expression analysis after vs. before treatment with tezepelumab in unstimulated HBECs, normalised to placebo. Volcano plot showing differentially expressed genes in red (FDR < 0.1) **(A)** and rlog change in average S100A8 and S100A9 expression from before (V2) to after (V7) **(B)**. Relative change of differentially expressed genes S100A8 **(C)** and S100A9 **(D)** in unstimulated (control) or 10 μg/ml poly(I:C)-stimulated HBECs for 24 h (2^−ΔΔCt^, after–before) in the placebo (*n* = 13) and tezepelumab (*n* = 13). **(E, F)** S100A8 and S100A9 gene expression measured in HBECs (*n* = 8) pretreated with 2 ng/ml rTSLP for 1 h and stimulated, or not, with poly(I:C) for 24 h. Fold-change expression of S100A8 **(E)** and S100A9 **(F)** (2^−ΔCt^). An independent experiment was performed for each donor. Outliers were removed using ROUT (1%) analysis. Data are presented as mean ± SD.

Given the role of S100A8 and S100A9 in initiating and amplifying local inflammatory responses (4), these findings were validated by RT-qPCR in a larger cohort (tezepelumab *n* = 13, placebo *n* = 13), confirming a significant downregulation in unstimulated HBECs after tezepelumab treatment (**Figures 1C, D**). A similar trend was observed after poly(I:C) stimulation but did not reach significance. ELISA showed no significant changes in S100A8/S100A9 protein secretion (**Supplementary Figure S1**). To assess whether these changes were directly due to TSLP blockade, baseline HBECs from six to seven donors were pretreated with rTSLP for 1 h, followed by 24 h poly(I:C) stimulation. rTSLP increased poly(I:C)-induced S100A8 and S100A9 mRNA expression (**Figures 1E, F**). Together, these results demonstrate that TSLP modulates poly(I:C)-induced S100A8 and S100A9 expression, whilst tezepelumab treatment reduces the baseline expression in HBECs.

### Transcriptomic analysis revealed that TSLP blockade downregulates the NF-κB-mediated inflammatory signalling pathway in virus-stimulated HBECs

Our previous work demonstrated that tezepelumab reduces epithelial alarmin IL-33 and T2 cytokine release in response to poly(I:C) stimulation (15). Here, we aimed to elucidate the molecular pathways through which tezepelumab attenuates proinflammatory cytokine production in HBECs. Transcriptomic analysis identified 63 of 594 genes as DEGs (FDR < 0.1) in poly(I:C)-stimulated HBECs after tezepelumab treatment, 62 of which were downregulated and one upregulated (**Figure 2A**; **Supplementary Table S2**). ORA using MSigDB revealed a single pathway overrepresented, “TNF-α signalling via NF-κB” (**Figure 2A**; **Supplementary Figure S2**), a key regulator of proinflammatory mediators, including T2 cytokines.

**Figure 2:**
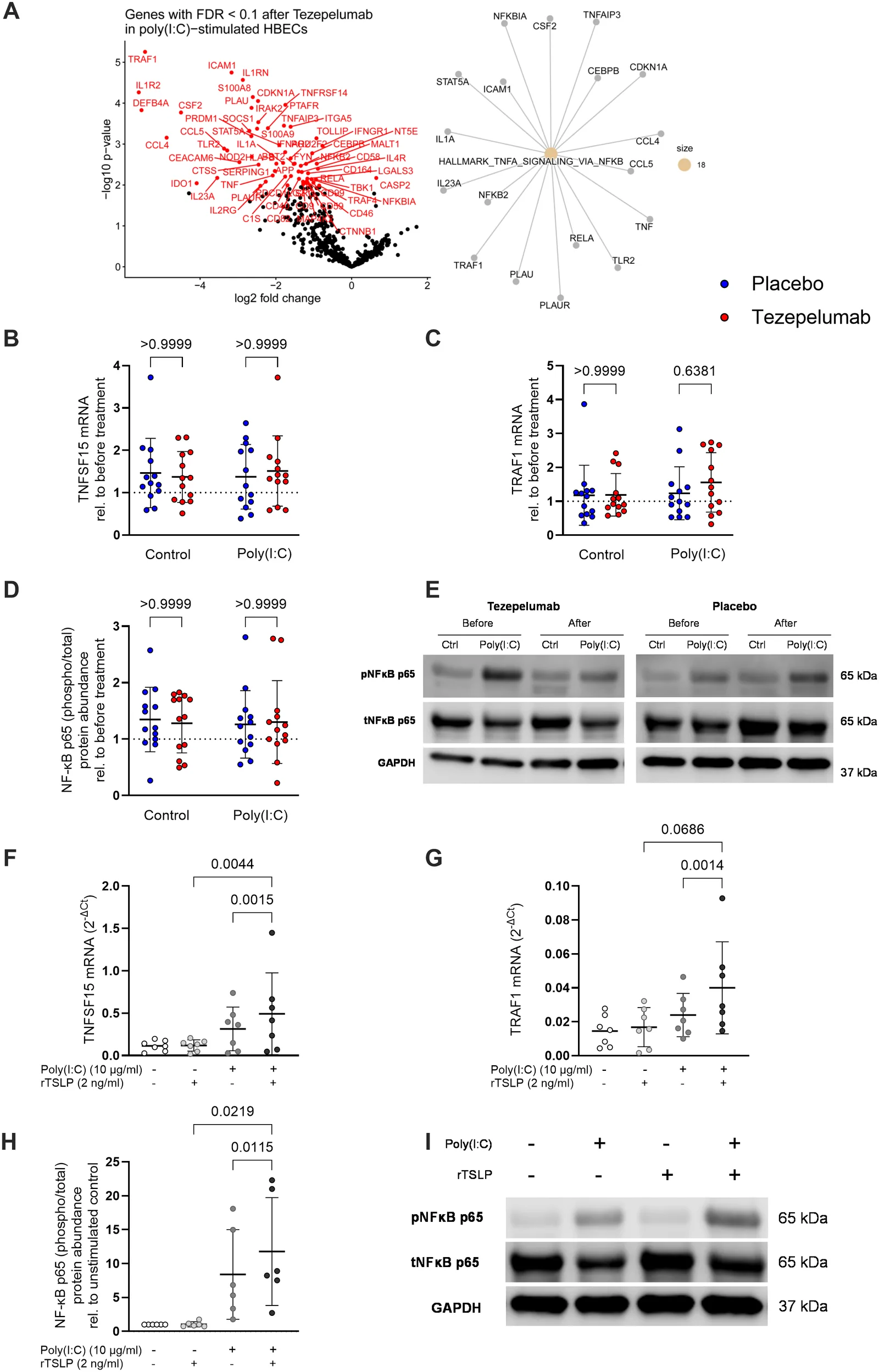
Effects of tezepelumab treatment and rTSLP on NF-kB inflammatory pathways in HBECs after viral stimulation. **(A)** Differential gene expression analysis before vs. after treatment with tezepelumab in poly(I:C)-stimulated HBECs normalised to placebo. Volcano plot showing differentially expressed genes in red (FDR < 0.1) (left) and a cnetplot including genes related to the hallmark pathway “TNF-a signalling via NF-kB” identified as downregulated after tezepelumab treatment (right). **(B, C)** Relative change of NF-kB-related genes TNFSF15 **(B)** and TRAF1 **(C)** in unstimulated (control) or 10 mg/ml poly(I:C)-stimulated HBECs for 24 h (2−DDCt, after–before) from placebo (n = 13) and tezepelumab (n = 13). **(D)** Relative change in phospho/total-NF-kB p65 protein abundance (after/before) with tezepelumab compared with placebo in unstimulated (control) or poly(I:C)-stimulated HBECs for 2 h and **(E)** representative Western blot images of pNF-kB p65. **(F, G)** NF-kB-related gene expression measured in HBECs (n = 8) pretreated with 2 ng/ml rTSLP for 1 h and stimulated with or without poly(I:C) for 24 h. Fold-change expression of TNFSF15 **(F)** and TRAF1 **(G)** (2−DCt). **(H, I)** Phospho-NF-kB p65/total NF-kB p65 protein ratio was measured in HBECs (n = 6) pretreated with 2 ng/ml rTSLP for 1 h and stimulated with or without poly(I:C) for 2 h. Fold-change of phospho-NF-kB p65/total NF-kB p65 protein ratio normalised to control **(H)** and representative Western blot image **(I)**. Data are presented as mean ± SD.

To further evaluate this effect of tezepelumab treatment on the NF-κB signalling pathway, we analysed the expression of TNFSF15 and TRAF1 (TNF receptor-associated factor 1) by RT-qPCR and the relative phosphorylation of the NF-κB p65 subunit (encoded by the *RELA* gene) by Western blotting in the full patient cohort (tezepelumab/*n* = 13, placebo/*n* = 13). TNFSF15 and TRAF1 were evaluated as representative NF-κB–responsive genes involved in inflammatory signalling, thereby serving as transcriptional readouts of NF-κB pathway activation and its modulation by tezepelumab. No significant differences were detected between placebo- and tezepelumab-treated groups in either unstimulated or poly(I:C)-stimulated HBECs. However, substantial heterogeneity in the expression levels of *TNFSF15*, *TRAF1*, and phosphorylated p65 was observed within the tezepelumab cohort (**Figures 2B–E**).

We next investigated whether TSLP directly contributes to NF-κB activation by treating HBECs (*n* = 6) with rTSLP, followed by stimulation with or without poly(I:C) for 24 h. rTSLP enhanced the activation of the NF-κB signalling pathway in response to poly(I:C) stimulation, as evidenced by the phosphorylation of NF-κB–related proteins (**Figures 2H, I**). These findings indicate that TSLP can directly amplify NF-κB activation during poly(I:C) stimulation and suggest that the transcriptional downregulation observed following tezepelumab treatment may reflect suppression of TSLP-mediated signalling.

### Tezepelumab-treated patients were stratified based on their baseline inflammatory profile

Given the heterogeneous NF-κB-related responses to tezepelumab in our cohort, we performed exploratory stratification of the study population. Using our previously published study data (15), we stratified tezepelumab-treated patients by a hierarchical cluster analysis (**Figure 3**) to classify donors according to the molecular phenotype of the bronchial epithelium. Considering the central role of the NF-κB pathway in driving inflammatory responses, we hypothesised that the observed variability could be explained by biological differences in the proinflammatory response to poly(I:C) prior to tezepelumab treatment, i.e., the molecular phenotype. For this analysis, we included baseline FeNO (ppb) levels together with poly(I:C)-induced levels of TNF-α, T2 cytokines (IL-4, IL-5, and IL-13), and the epithelial alarmin TSLP measured in HBECs collected before tezepelumab treatment.

**Figure 3:**
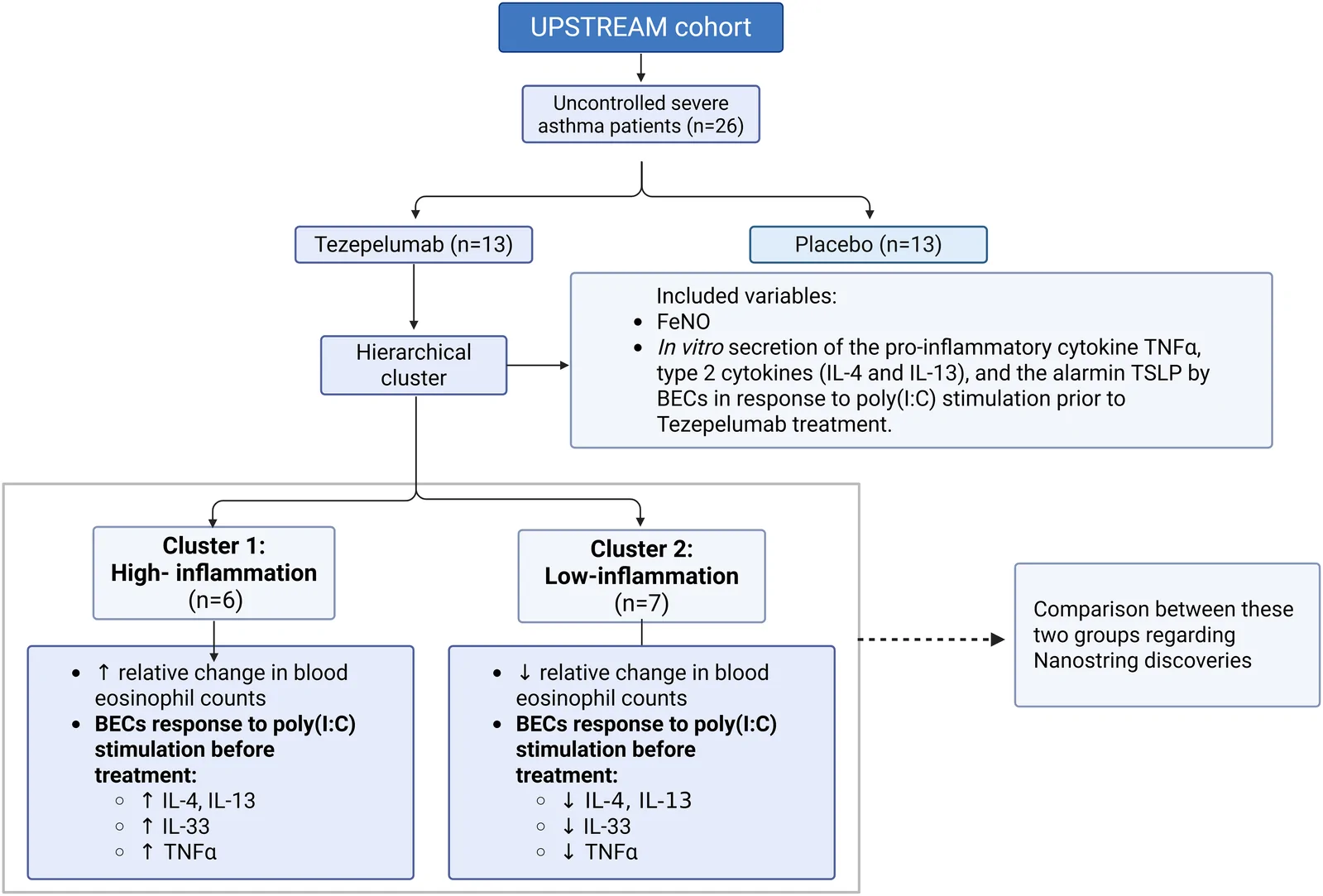
Flowchart illustrating the stratification and clustering of patients from the UPSTREAM cohort based on inflammation-related variables before tezepelumab treatment. HBECs, human bronchial epithelial cells.

Two distinct clusters were identified (average silhouette width = 0.34) (**Figure 3**, **4A**): cluster 1 (high-inflammation), characterised by a greater relative change in eosinophil counts (after/before) and elevated IL-4, IL-13, IL-33, and TNF-α in response to poly(I:C) stimulation compared with cluster 2 (low-inflammation) (**Supplementary Table S3**). Although the two groups were similar with respect to lung function- and asthma control-related characteristics prior to tezepelumab treatment (**Supplementary Table S4**), the “high-inflammation” group tended to have higher levels of T2 biomarkers such as blood eosinophil counts, FeNO, and serum IgE. This stratification enabled us to evaluate molecular responses to tezepelumab according to baseline inflammatory status.

**Figure 4:**
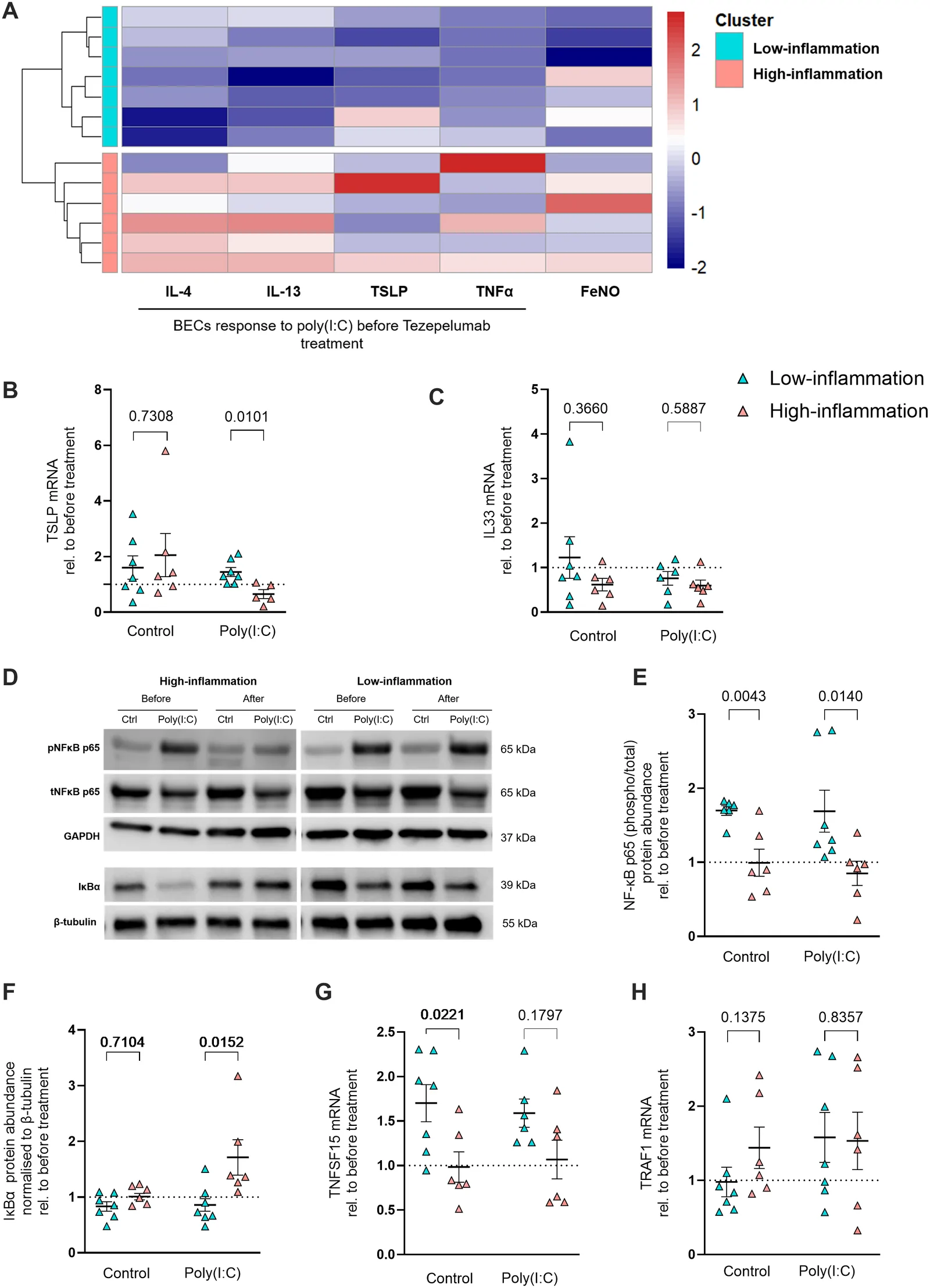
Patient stratification of the tezepelumab cohort based on inflammatory markers prior to treatment. **(A)** Heatmap showing hierarchical clustering of tezepelumab patients in low-inflammation (turquoise) and high-inflammation (salmon) groups. Values for all included variables were scaled by row (*Z*-score normalisation). Red indicates higher values, and blue indicates lower ones. **(B)** Relative change in alarmin mRNA expression of TSLP **(B)** and IL-33 **(C)** in unstimulated (control) or 10 μg/ml poly(I:C)-stimulated HBECs for 24 h (2^−ΔΔCt^, after–before) from low-inflammation (*n* = 7) and high-inflammation (*n* = 6). **(D)** Representative Western blot images of phospho-NF-κB p65 and IκBα. **(E)** Relative change in phospho-NF-κB p65 protein abundance (after/before) in low-inflammation compared with high-inflammation in unstimulated (control) or 10 μg/ml poly(I:C)-stimulated HBECs for 2 h. **(F)** Relative change in IκBα protein abundance (after/before) in low-inflammation compared with high-inflammation in unstimulated (control) or 10 μg/ml poly(I:C)-stimulated HBECs for 2 h. **(G, H)** Relative change of NF-κB-related mRNA expression of TNFSF15 **(G)** and TRAF1 **(H)** in unstimulated (control) or 10 μg/ml poly(I:C)-stimulated HBECs for 24 h (2^−ΔΔCt^, after–before) from low-inflammation (*n* = 7) and high-inflammation (*n* = 6). Outliers were removed using ROUT (1%) analysis. Data are presented as mean ± SD.

### Baseline inflammatory profile defines distinct molecular responses to tezepelumab treatment

Patient stratification revealed a significant difference in TSLP fold-change (after–before) between clusters, with the high-inflammation group showing a reduction (fold-change < 1) and the “low-inflammation” group showing an increase (fold-change > 1) (**Figure 4B**). This difference was observed only for TSLP at the gene level, with no significant difference in IL-33 expression (**Figure 4C**). Notably, the high-inflammation group exhibited lower levels of the phosphorylated p65 subunit in both unstimulated and poly(I:C)-stimulated conditions and increased levels of IκBα inhibitor after tezepelumab treatment only in poly(I:C)-stimulated HBECs compared with the low-inflammation group (**Figures 4D–F**; **Supplementary Figure S3**). A similar trend was observed for TNFSF15 (**Figure 4G**), but not for TRAF1 (**Figure 4H**). Additionally, a significant correlation was observed between the change in NF-κB p65 activation and the change in blood eosinophil counts following tezepelumab treatment under unstimulated conditions (Pearson’s *r* = 0.893, 95% confidence interval [CI]: 0.654–0.970, *R*^2^ = 0.797, *p* < 0.0001; *n* = 12; **Supplementary Figure S4A**). Similarly, a significant correlation was observed between the change in IκBα and blood eosinophil counts under unstimulated conditions (*r* = − 0.627, 95% CI: − 0.875 to − 0.115, *R*^2^ = 0.393, *p* = 0.022; *n* = 13; **Supplementary Figure S4B**). In contrast, no significant correlation was observed under poly(I)-stimulated conditions. Altogether, these data indicate a differential molecular response to tezepelumab treatment according to the baseline inflammatory profile of patients.

### Tezepelumab influences the epithelial–smooth muscle cell crosstalk via downregulation of TGF-b2 in BSMCs

Bronchial epithelial–smooth muscle interactions in asthma create a feedback loop in which epithelium-derived mediators (e.g., TSLP) drive smooth muscle proliferation and airway remodelling (18). Previously, we showed that CM from unstimulated and poly(I:C)-stimulated HBECs increased IL-33 gene and protein expression in BSMCs (11). To assess whether tezepelumab modulates this crosstalk, we analysed transcriptomic changes in HBECs related to airway remodelling. We selected six GO terms associated with key remodelling processes: TGF-β signalling (GO:0007179), extracellular matrix organisation (GO:0030198), cell migration (GO:0016477), cell proliferation (GO:0008283), angiogenesis (GO:0001525), and response to hypoxia (GO:0001666). For each category, we assessed the mean expression of relevant genes (before and after) normalised to placebo. We found that tezepelumab treatment induces downregulation of genes related to airway remodelling in both unstimulated (**Supplementary Figure S5**) and poly(I:C)-stimulated (**Figures 5A, B**) HBECs.

**Figure 5:**
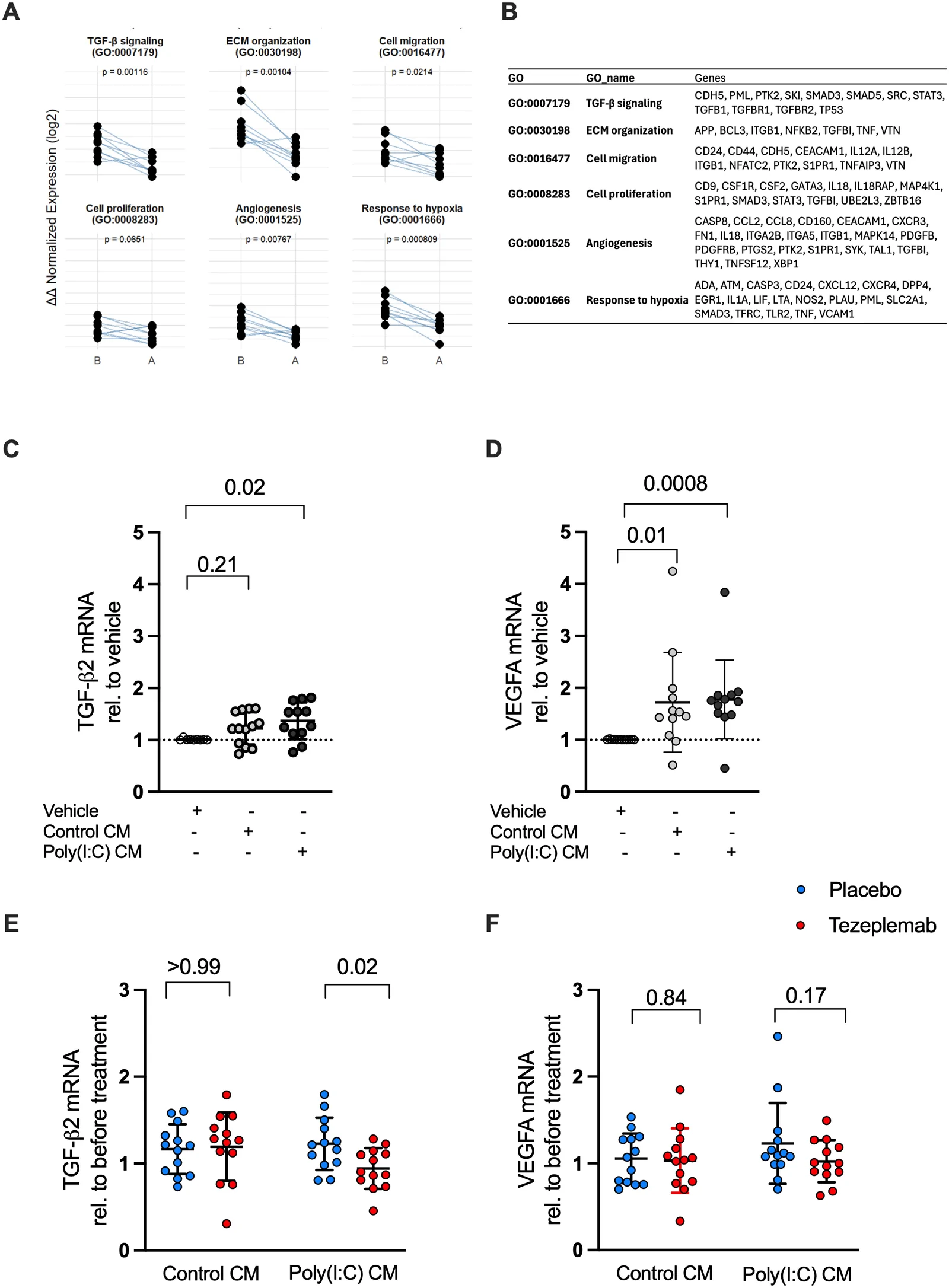
Tezepelumab influences epithelial–smooth muscle cell crosstalk via downregulation of growth factors in BSMCs. **(A)** Mean normalised expression (before and after) relative to placebo of genes included in six different Gene Ontology (GO) terms associated with airway remodelling processes. **(B)** Genes included in each GO term category. **(C–F)** BSMCs were treated with conditioned media (CM) at a 1:10 dilution from unstimulated and poly(I:C)-stimulated (24 h) HBECs treated with placebo (*n* = 13) or tezepelumab (*n* = 13). **(C)** Fold change in gene expression of TGF-β2. **(D)** Fold change in gene expression of VEGFA. **(E)** Fold change in gene expression of TGF-β2 after tezepelumab compared with placebo (2^−ΔΔCt^, after–before). **(F)** Fold change in gene expression of VEGFA after tezepelumab compared with placebo (2^−ΔΔCt^, after–before). Outliers were removed using ROUT (1%) analysis. Data are presented as mean ± SD.

To evaluate downstream effects, primary healthy BSMCs were treated *in vitro* with CM derived from either unstimulated or poly(I:C)-stimulated HBECs, collected before and after tezepelumab or placebo treatment. We first confirmed the effects of CM derived from both unstimulated and poly(I:C)-stimulated HBECs (before treatment) on gene expression in BSMCs. Our results demonstrated that HBEC CM significantly increased VEGFA expression in BSMCs under both experimental conditions. In contrast, a significant upregulation of TGF-β2 expression was observed only following exposure to CM from poly(I:C)-stimulated HBECs after 24 h (**Figures 5C, D**). We then compared TGF-β2 and VEGFA gene expression in BSMCs stimulated with CM from poly(I:C) stimulated HBECs obtained after *in vivo* tezepelumab or placebo treatment and found reduced TGF-β2 gene expression and a trend towards reduced VEGFA expression in BSMCs following tezepelumab treatment compared with placebo (**Figures 5E, F**).

## Discussion

In this study, using an exploratory multiplex gene expression analysis, we identified S100A8 and S100A9 as downregulated after tezepelumab treatment in both unstimulated and poly(I:C)-stimulated HBECs compared with placebo. Additional ORA suggested that tezepelumab reduced the enrichment of the TNF-α signalling via NF-κB hallmark pathway in poly(I:C)-stimulated HBECs. As this finding emerged from exploratory transcriptomic analyses, we sought to further investigate this pathway. Recombinant TSLP enhanced poly(I:C)-induced S100A8, S100A9, and NF-κB p65 phosphorylation in HBECs, indicating that TSLP can amplify these inflammatory responses. However, these gain-of-function experiments do not establish that inhibition of this pathway mediates the clinical effects of tezepelumab. Interestingly, six GO terms related to airway remodelling were also found to be downregulated after tezepelumab treatment. When examining the impact of bronchial epithelial mediators on BSMCs, we observed a significant reduction in the growth factor TGF-β2 and a trend towards decreased VEGFA levels in BSMCs following tezepelumab treatment compared with placebo. These results suggest that tezepelumab may attenuate virus-induced proinflammatory responses and modulate airway proremodelling genes, both of which are key contributors to asthma exacerbations.

S100A8 and S100A9 are known to function as damage-associated molecular patterns (DAMPs), initiating and amplifying local inflammatory responses (19, 20). These proteins are implicated in the pathogenesis of chronic inflammatory diseases, including asthma, where aberrant regulation of S100 proteins is correlated with disease persistence and severity (21–23), especially in neutrophilic asthma (22–24). In fact, S100A8 or S100A9 are upregulated in HBECs from patients with severe asthma compared to controls (25), and their knockdown inhibited airway inflammation in OVA-sensitised mice (26).

Although S100 proteins are known to display antibacterial properties (27), they also participate in antiviral immune responses (28). Our *in vitro* observations showed that S100A8 and S100A9 mRNA levels were increased after viral mimic poly(I:C) stimulation, and the addition of rTSLP further increased their expression levels. This aligns with prior studies showing that S100A8 and S100A9 amplify *Alternaria*-induced production of TSLP and IL-25 (29), suggesting a feedback loop between S100 proteins and classical alarmins. Altogether, our observations suggest that tezepelumab treatment could attenuate the positive feedback loop contributing to an excessive proinflammatory response to viral infection driving asthma exacerbations, whilst not affecting antiviral proteins, as previously observed in our cohort (15).

Building on our previous findings that tezepelumab reduces IL-33 and T2 cytokines following viral challenge, here we investigated the underlying molecular pathways. Our ORA suggested a downregulation of the TNF-α signalling via the NF-κB hallmark pathway. Importantly, this observation was not confirmed in the overall validation analyses of TNFSF15, TRAF1, or phosphorylated NF-κB p65/IκBα in the full cohort. Instead, substantial inter-individual variability was observed. To further investigate this variability, we performed an exploratory clustering analysis based on molecular markers of epithelial inflammation in response to poly(I:C) prior to tezepelumab treatment, using data from our previously published study (15). In addition, baseline FeNO levels were included as a biomarker of local airway inflammation, as HBECs are the primary source of nitric oxide (NO) in the airways (30). These subgroup analyses should therefore be regarded as hypothesis-generating rather than confirmatory. Within this exploratory framework, the stratification analysis suggested that patients with uncontrolled asthma and higher baseline inflammation exhibited reduced NF-κB pathway activation in response to the viral mimic following tezepelumab treatment. Our correlation analysis further highlighted an association between the magnitude of NF-κB pathway activation and the change in blood eosinophil counts following tezepelumab treatment under unstimulated conditions, but not following poly(I:C) stimulation. The link between this molecular pathway and T2 mediators is of particular interest. On the one hand, IL-33 induces activation of the NF-κB molecular pathway on multiple cell types (31, 32). In contrast, receptors for T2 cytokines (IL-4 and IL-13) primarily signal through the JAK/STAT pathway, although NF-κB has also been described as being required (33, 34). At the same time, the activation of this signalling pathway is needed for the expression of proinflammatory mediators. Particularly, the TLR4/NF-κB signalling pathway is triggered by allergens and is needed to start the IL-33/ST2/T2 response (35). In addition, a novel treatment ameliorates AHR via downregulation of the IKK/NF-κB/IL-4/IL-5/IL-13 signalling pathway (36). Other T2 biomarkers, including FeNO, are also dependent on this signalling pathway. NO production is mediated by inducible NO synthase (iNOS), whose expression is regulated by several signalling pathways, including NF-κB (37). Altogether, these observations suggest the possibility that the NF-κB molecular pathway contributes to the modulation of IL-33 and T2 cytokines (IL-4 and IL-13) in response to viral stimulation (15) following TSLP blockade in a subset of patients with high basal inflammation. However, because these observations were not confirmed in the overall validation analyses and the subgroup analyses were performed *post hoc*, this proposed mechanism should be considered exploratory. Other molecular pathways may also contribute to the observed effects of tezepelumab, and larger studies will be required to determine the role of NF-κB signalling.

Interestingly, the reduction of TSLP expression was observed only in the high-inflammation cluster following poly(I:C) stimulation, a group that also exhibited higher activation of NF-κB–related mediators and a greater reduction in phosphorylated p65 after tezepelumab treatment. Mechanistically, poly(I:C) stimulation activates TLR3-dependent antiviral signalling in HBECs, leading to activation of NF-κB and induction of inflammatory mediators (38). In contrast, IL-33 expression following poly(I:C) stimulation was reduced across both clusters, indicating that its regulation in the context of antiviral epithelial responses may occur independently of the baseline level of NF-κB activation. These findings may therefore reflect differential regulation of epithelial alarmins during viral sensing, with TSLP induction being more dependent on inflammatory amplification pathways, whereas IL-33 modulation may occur through broader epithelial antiviral signalling mechanisms. However, these interpretations remain exploratory and should be confirmed in larger cohorts.

The observed epithelial changes likely influence downstream immune and structural cell responses, particularly in BSMCs, which are central to asthma pathophysiology and airway remodelling. Previously, we showed that tezepelumab improves AHR in response to mannitol (13) and modulates the release of proinflammatory mediators from poly(I:C)-stimulated HBECs (15). Therefore, here we aimed to investigate how these changes in epithelial proinflammatory mediators could influence underlying BSMC biology. Our transcriptomic analysis showed that HBECs express lower levels of proremodelling mediators after tezepelumab treatment compared with before treatment. The effects of anti-TSLP on improving asthma remodelling have already been described. For instance, anti-TSLP reduces the levels of MMP and CTGF in BALF (39) and inhibits the structural changes associated with airway remodelling (40) in murine models. In support of these previous findings, we show that BSMCs stimulated with CM from HBECs following tezepelumab treatment exhibit reduced expression of the mitogenic growth factor TGF-β2 compared with placebo. A similar trend was observed for VEGFA. TGF-β promotes BSMC proliferation and induces the expression of other growth factors such as VEGF (41). TGF-β2, predominantly produced by alveolar type I, endothelial, and smooth muscle in the lung (https://www.proteinatlas.org/ENSG00000092969-TGFB2/single+cell/lung), is elevated in severe compared to mild asthma (42) and promotes MUC5AC overexpression in HBECs (43). Moreover, VEGF induces the migration of BSMCs (44), another proremodelling feature described in asthma. Together, these findings suggest that epithelial-derived mediators following tezepelumab treatment can influence the expression of remodelling-associated transcripts in BSMCs in a conditioned-medium *in vitro* model. Whether these transcriptional changes translate into altered BSMC proliferation, migration, or airway remodelling *in vivo* remains unknown. Further studies incorporating functional and structural remodelling endpoints are needed to determine the biological significance of these observations.

The main strength of this study is the use of primary HBECs from patients with uncontrolled asthma who had received tezepelumab in clinical practice, capturing both direct TSLP blockade and reduced local inflammation after 12 weeks. However, several limitations exist. First, HBECs were cultured as submerged monolayers rather than differentiated air–liquid interface (ALI) cultures, which may not fully recapitulate the cellular diversity and physiological characteristics of the airway epithelium, particularly in response to viral infection. Second, poly(I:C), a synthetic analogue of viral dsRNA, was used to mimic viral stimulation. Whilst it reproduces key features of RV-induced innate immune activation with high reproducibility, it avoids confounding effects of viral replication-associated cytotoxicity (6). Third, the small NanoString sample size may limit statistical power; although a larger validation cohort (tezepelumab *n* = 13, placebo *n* = 13) confirmed gene expression trends, not all reached significance, underscoring the need for larger studies. *Post hoc* hierarchical clustering identified two patient subgroups, but moderate overlap, as indicated by the silhouette width, indicates that these findings are exploratory and hypothesis-generating, particularly regarding differential NF-κB-related responses. This heterogeneity further supports the need for larger cohorts. Finally, *in vitro* experiments using exogenous rTSLP confirmed a direct role of TSLP in modulating S100A8/A9 expression and NF-κB pathway activation during the anti-inflammatory response, strengthening the findings. Overall, despite limitations, the exploratory design and use of primary human samples provide strong translational and clinical relevance.

In conclusion, our study demonstrates that treatment of patients with uncontrolled asthma with tezepelumab induces transcriptomic changes in the HBECs. We found that tezepelumab treatment led to decreased expression of S100A8 and S100A9 in both unstimulated and poly(I:C)-stimulated HBECs and exploratory transcriptomic evidence suggesting altered NF-κB-related signalling in response to poly(I:C) stimulation. These transcriptomic alterations were associated with changes in the expression of genes related to remodelling processes in downstream BSMCs. Overall, these findings generate hypotheses regarding the molecular pathways through which TSLP blockade may modulate epithelial inflammatory responses and their downstream effects, which warrant further mechanistic investigation.

## Data Availability

The datasets presented in this study can be found in online repositories. The names of the repository/repositories and accession number(s) can be found below: GSE336073 (GEO).
