# Supplementary material for: Tezepelumab alters airway epithelial transcriptional responses and epithelial–smooth muscle signalling in uncontrolled asthma
Source: Front Immunol. 2026 Oct 16;17:1911911. doi: 10.3389/fimmu.2026.1911911 (PMC13719334; doi:10.3389/fimmu.2026.1911911)
Supplement: Supplementary file 1 [file DataSheet1.pdf]

***Tezepelumab alters airway epithelial transcriptional responses and epithelial–smooth muscle signalling in uncontrolled asthma***

*Short title: Tezepelumab Modulates Airway Epithelial Inflammation*

Authors: S Ramu[1], S Vázquez-Mera[1,] M Menzel[1], S Cerps[1], JJ Nieto-Fontarigo[1,2,3,4], M Hvidtfeldt[5], C Porsbjerg[5], A Sverrild[5*] and L Uller[1*]

*1. Unit of Respiratory Immunopharmacology, Department of Experimental Medical Science, Lund University, Lund, Sweden.*
*2. BioLympho Research Group, Department of Biochemistry and Molecular Biology, Faculty of Biology-Biological Research Centre (CIBUS), University of Santiago de Compostela, Santiago de Compostela, Spain.*
*3. Translational Research in Airway Diseases Group (TRIAD), Health Research Institute of Santiago de Compostela (IDIS), Santiago de Compostela, Spain.*
*4. Aquatic One Health Research Center (ARCUS), Universidade de Santiago de Compostela, Santiago de Compostela, Spain.*
*5. Department of Respiratory Medicine, University Hospital Bispebjerg, Copenhagen, Denmark.*

Corresponding author: lena.uller@med.lu.se

Keywords: Tezepelumab; asthma; alarmins; transcriptomics; epithelium; smooth muscle

# Supplementary Figures

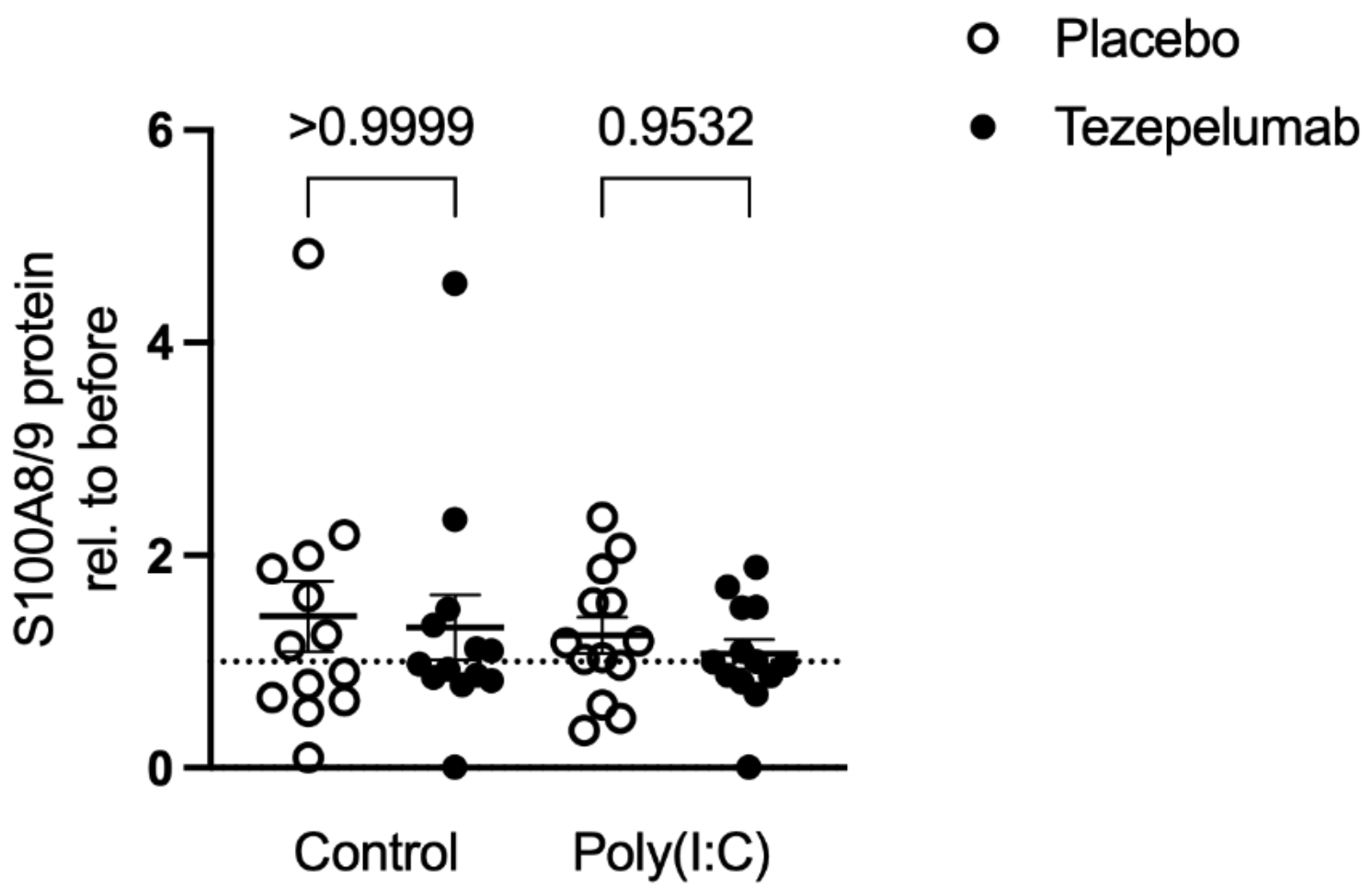


**Supplementary Figure 1. Fold-change of S100A8/9 heterodimer protein released by human bronchial epithelial cells (HBCs) after Tezepelumab or Placebo.** Relative change of S100A8/9 heterodimer abundance in the supernatant of unstimulated (control) or poly (I:C)-stimulated HBECs for 24 h (after/before) from Placebo (n=13) and Tezepelumab (n=13).

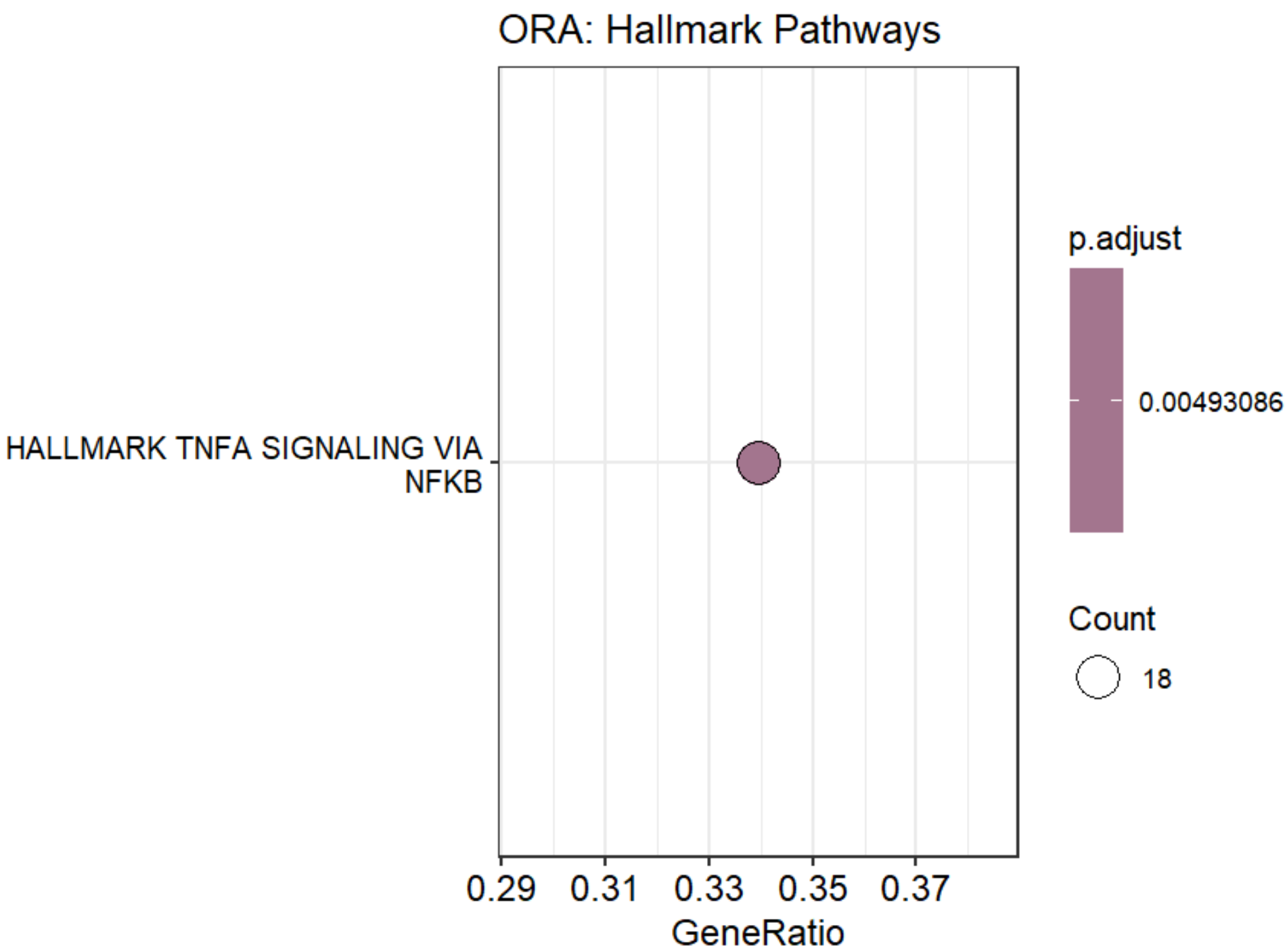


**Supplementary Figure 2. Over-representation Analysis (ORA) of downregulated Molecular Signatures Pathways after Tezepelumab treatment normalized to Placebo**. Molecular Signatures Database (MSigDB) and the R package msigdbr were used.

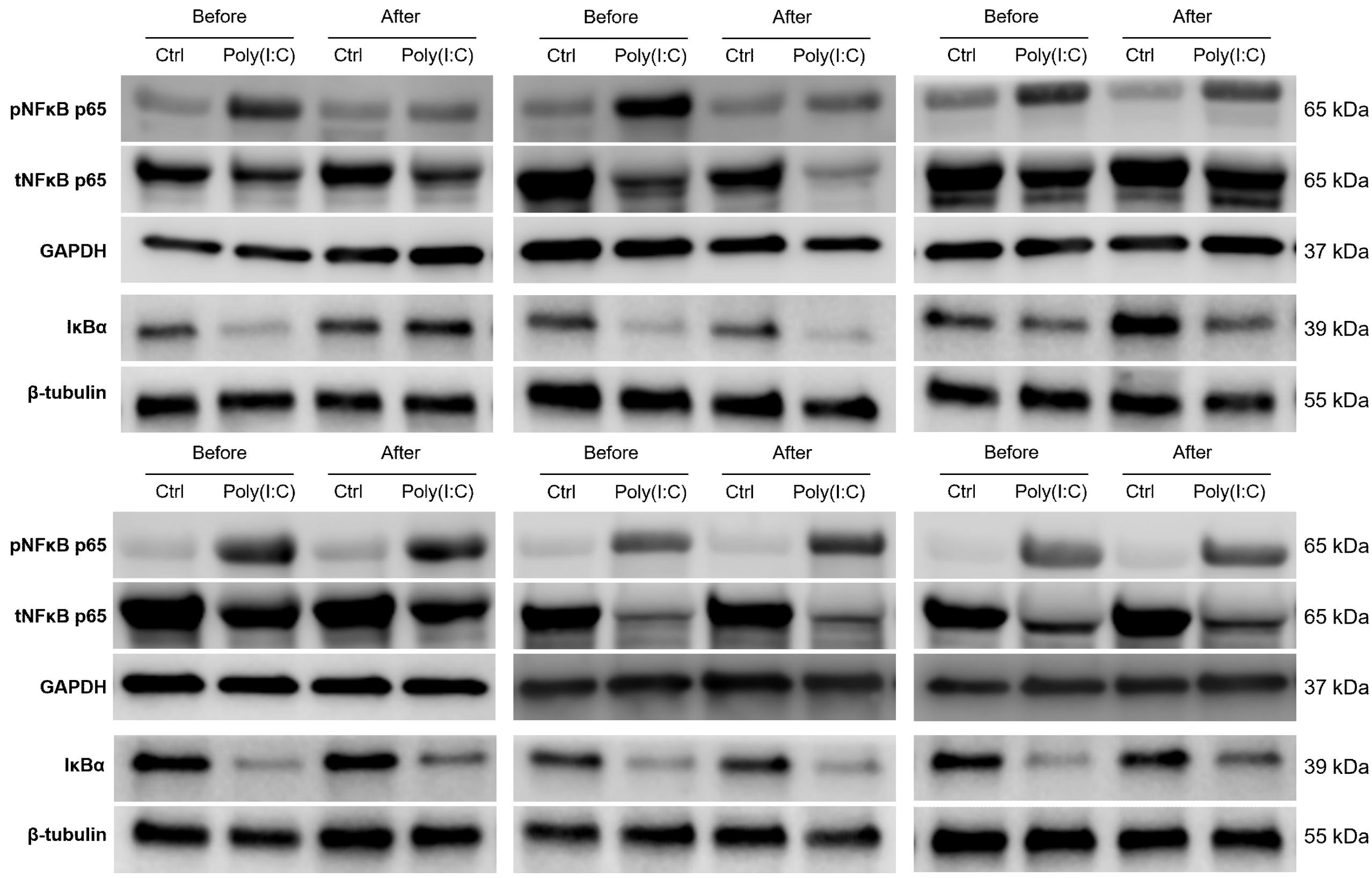


**Supplementary Figure 3. Western blot images showing the decreased phosphorylation of the NFκB p65 subunit and IκBα in the "high-inflammation" cluster (n=6) after Tezepelumab treatment.** Relative changes in phosphorylated NF-κB p65 (pNF-κB p65) and IκBα were quantified in unstimulated (Ctrl) and poly(I:C)-stimulated HBECs after 2 h within the 'high-inflammation' cluster (after/before treatment). pNF-κB p65 values were normalised to total NF-κB p65 (tNF-κB p65), with GAPDH serving as a loading control. IκBα was normalised using β-tubulin. The first western blot represents a donor included as a representative blot in Figure 4.

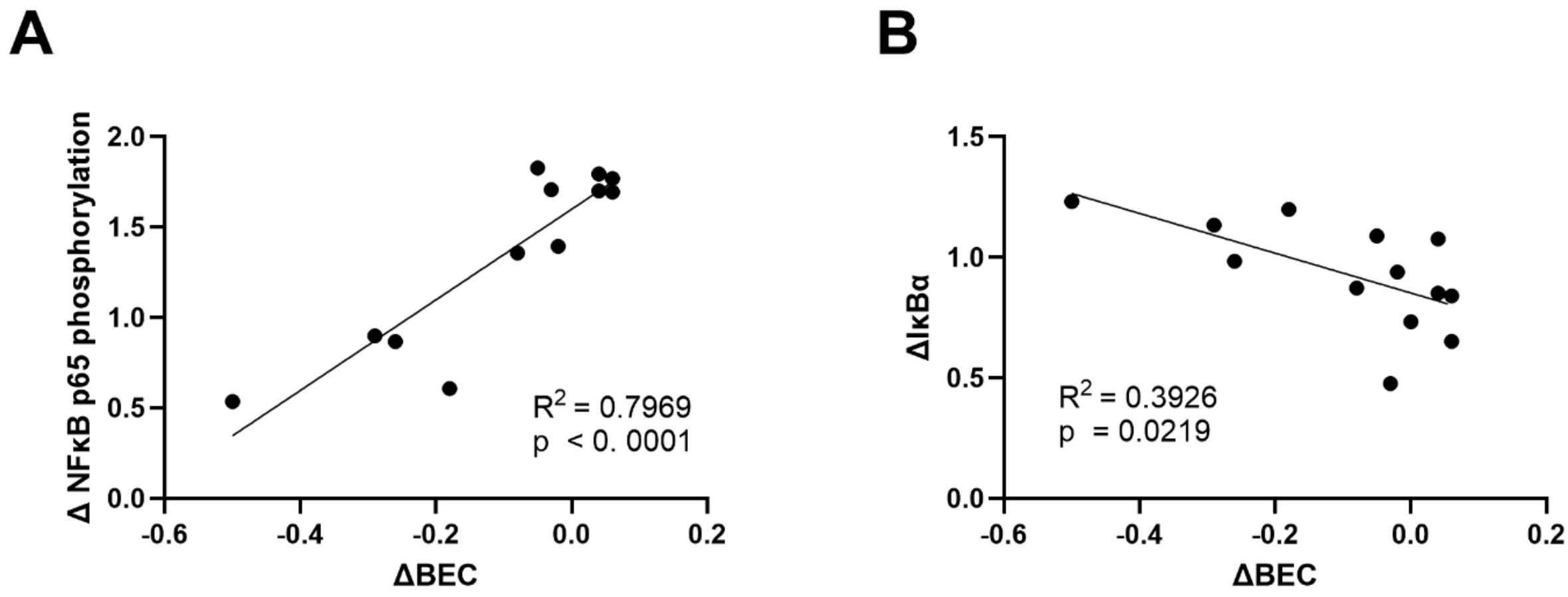


**Supplementary Figure 4. Correlation between changes in NF-κB p65 phosphorylation, IκBα, and blood eosinophil counts following tezepelumab treatment under unstimulated conditions.** (A) Correlation between the change in NF-κB p65 phosphorylation and the change in blood eosinophil counts (ΔBEC) under unstimulated conditions ($r = 0.893$, $R^2 = 0.797$, $P < 0.0001$, $n = 12$). (B) Correlation between the change in IκBα (ΔIκBα) and ΔBEC under unstimulated conditions ($r = -0.627$, $R^2 = 0.393$, $P = 0.0219$, $n = 13$). Each dot represents an individual patient. Lines indicate linear regression. Pearson correlation analysis was performed.

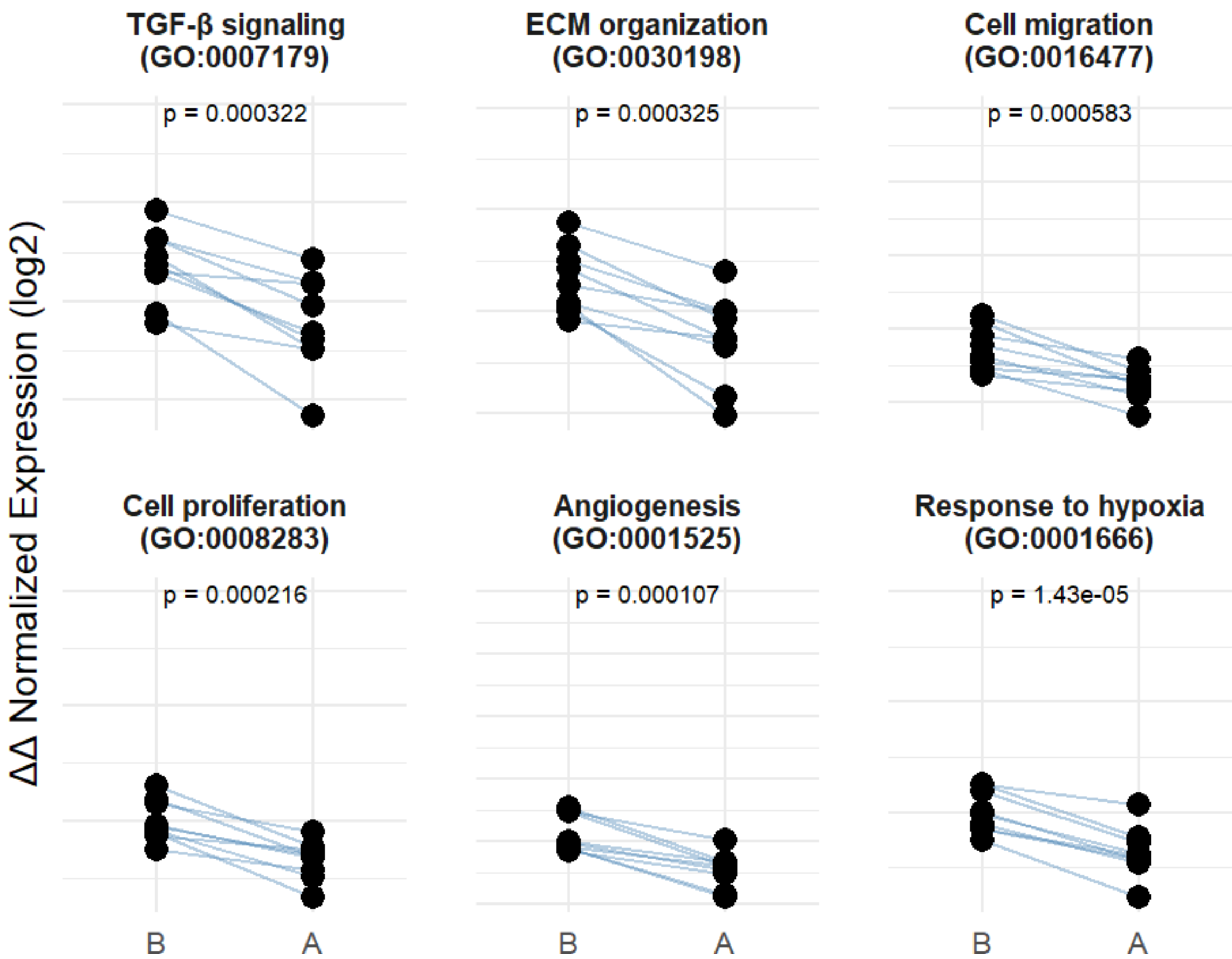


**Supplementary Figure 5. Gene Ontology (GO) terms related with airway remodelling downregulated after Tezepelumab in unstimulated conditions normalized with Placebo group.** To identify genes included in specific Gene Ontology (GO) terms related to airway remodelling processes, Genome wide annotation for Human database and the "org.Hs.eg.db" R package were employed. Abbreviations: B (before) A (after) tezepelumab treatment. Significant differences were tested using paired t-tests.

# Supplementary Tables

**Supplementary Table 1. Differentially expressed genes after vs. before Tezepelumab treatment on unstimulated HBECs.** Differential expression analysis using DESeq2 (~ Visit + Treatment + Visit:Treatment)

| | baseMean | log2FC | lfcSE | stat | p-value | Padj |
|---|---|---|---|---|---|---|
| S100A8 | 7337.494 | -2.973 | 0.668 | -4.450 | 0.000009 | 0.002547 |
| S100A9 | 14697.494 | -2.829 | 0.619 | -4.568 | 0.000005 | 0.002547 |

**Supplementary Table 2. Differentially expresed genes after vs. before Tezepelumab treatment on poly(I:C)-stimulated HBECs.** Differential expression analysis using DESeq2 (~ Visit + Treatment + Visit:Treatment)

| | baseMean | log2FC | lfcSE | stat | p-value | padj |
|---|---|---|---|---|---|---|
| IL1R2 | 3809.007 | -5.618 | 1.392 | -4.036 | 0.00005 | 0.00806 |
| DEFB4A | 5971.919 | -5.546 | 1.462 | -3.794 | 0.00015 | 0.00977 |
| TRAF1 | 763.320 | -5.452 | 1.200 | -4.542 | 0.00001 | 0.00331 |
| CCL4 | 446.541 | -4.883 | 1.440 | -3.390 | 0.00070 | 0.02255 |
| CSF2 | 4094.488 | -4.504 | 1.198 | -3.761 | 0.00017 | 0.01003 |
| IDO1 | 3169.924 | -4.090 | 1.568 | -2.609 | 0.00907 | 0.09275 |
| IL23A | 917.367 | -3.548 | 1.309 | -2.710 | 0.00673 | 0.08594 |
| CCL5 | 14471.123 | -3.366 | 1.046 | -3.218 | 0.00129 | 0.03476 |
| TLR2 | 1732.087 | -3.285 | 1.030 | -3.188 | 0.00143 | 0.03694 |
| ICAM1 | 7033.412 | -3.170 | 0.739 | -4.290 | 0.00002 | 0.00529 |
| CEACAM6 | 1580.313 | -2.963 | 0.990 | -2.993 | 0.00276 | 0.05346 |
| IL1RN | 9983.801 | -2.872 | 0.684 | -4.198 | 0.00003 | 0.00533 |
| SOCS1 | 1291.168 | -2.702 | 0.773 | -3.494 | 0.00048 | 0.01763 |
| PRDM1 | 1217.250 | -2.647 | 0.776 | -3.413 | 0.00064 | 0.02240 |
| PLAU | 26999.815 | -2.643 | 0.691 | -3.825 | 0.00013 | 0.00968 |
| NOD2 | 165.438 | -2.637 | 0.855 | -3.084 | 0.00204 | 0.04662 |
| S100A8 | 24659.801 | -2.608 | 0.656 | -3.973 | 0.00007 | 0.00842 |
| S100A9 | 46895.879 | -2.495 | 0.708 | -3.526 | 0.00042 | 0.01666 |
| CDKN1A | 8799.167 | -2.471 | 0.630 | -3.920 | 0.00009 | 0.00876 |
| IRAK2 | 1599.908 | -2.463 | 0.680 | -3.622 | 0.00029 | 0.01573 |
| CTSS | 5113.205 | -2.457 | 0.833 | -2.949 | 0.00319 | 0.05402 |
| PLAUR | 1742.685 | -2.407 | 0.940 | -2.559 | 0.01048 | 0.09868 |
| IL2RG | 132.254 | -2.267 | 0.859 | -2.640 | 0.00829 | 0.09161 |
| PTAFR | 998.002 | -2.210 | 0.625 | -3.534 | 0.00041 | 0.01666 |
| TNF | 469.589 | -2.084 | 0.756 | -2.755 | 0.00588 | 0.08296 |
| SERPING1 | 1015.652 | -2.027 | 0.715 | -2.837 | 0.00456 | 0.07116 |
| HLA-B | 13146.207 | -1.987 | 0.670 | -2.966 | 0.00301 | 0.05346 |
| STAT5A | 154.808 | -1.909 | 0.583 | -3.275 | 0.00106 | 0.03118 |
| IL1A | 13647.147 | -1.810 | 0.608 | -2.979 | 0.00289 | 0.05346 |
| TNFAIP3 | 7015.679 | -1.787 | 0.500 | -3.572 | 0.00035 | 0.01666 |
| C1S | 754.501 | -1.754 | 0.641 | -2.735 | 0.00624 | 0.08416 |
| IFNAR2 | 759.100 | -1.754 | 0.555 | -3.161 | 0.00157 | 0.03888 |
| TNFRSF14 | 228.536 | -1.749 | 0.452 | -3.867 | 0.00011 | 0.00935 |
| CD40 | 541.767 | -1.693 | 0.659 | -2.569 | 0.01021 | 0.09868 |
| ITGA5 | 5940.135 | -1.610 | 0.453 | -3.556 | 0.00038 | 0.01666 |
| POU2F2 | 343.871 | -1.609 | 0.526 | -3.058 | 0.00223 | 0.04899 |
| PDCD1LG2 | 634.972 | -1.602 | 0.584 | -2.745 | 0.00605 | 0.08343 |
| BST2 | 2001.140 | -1.529 | 0.516 | -2.961 | 0.00307 | 0.05346 |
| FYN | 171.001 | -1.503 | 0.507 | -2.967 | 0.00301 | 0.05346 |
| APP | 12075.070 | -1.401 | 0.495 | -2.828 | 0.00469 | 0.07133 |
| CD82 | 602.067 | -1.389 | 0.516 | -2.692 | 0.00709 | 0.08594 |
| NFKB2 | 1261.860 | -1.342 | 0.458 | -2.929 | 0.00340 | 0.05602 |
| CD58 | 1075.158 | -1.316 | 0.467 | -2.819 | 0.00482 | 0.07147 |
| SRC | 1141.680 | -1.283 | 0.486 | -2.638 | 0.00834 | 0.09161 |

*(Continued next page)*

**Supplementary Table 2 - continued. Differentially expresed genes after vs. before Tezepelumab treatment on poly(I:C)-stimulated HBECs.** Differential expression analysis using DESeq2 (~ Visit + Treatment + Visit:Treatment)

| | baseMean | log2FC | lfcSE | stat | p-value | padj |
|---|---|---|---|---|---|---|
| CD9 | 4018.835 | -1.278 | 0.493 | -2.591 | 0.00958 | 0.09630 |
| NT5E | 10958.533 | -1.204 | 0.406 | -2.963 | 0.00305 | 0.05346 |
| MAP4K2 | 228.026 | -1.201 | 0.459 | -2.617 | 0.00888 | 0.09234 |
| CD46 | 2850.523 | -1.183 | 0.447 | -2.648 | 0.00809 | 0.09161 |
| LGALS3 | 4065.010 | -1.168 | 0.433 | -2.696 | 0.00701 | 0.08594 |
| CD59 | 15270.747 | -1.161 | 0.444 | -2.617 | 0.00886 | 0.09234 |
| CD164 | 2448.865 | -1.150 | 0.411 | -2.796 | 0.00518 | 0.07494 |
| CTNNB1 | 9836.588 | -1.096 | 0.428 | -2.561 | 0.01045 | 0.09868 |
| NFKBIA | 7606.617 | -1.059 | 0.394 | -2.686 | 0.00723 | 0.08594 |
| CEBPB | 1679.735 | -1.041 | 0.331 | -3.143 | 0.00167 | 0.03970 |
| CD99 | 6132.260 | -1.007 | 0.384 | -2.621 | 0.00878 | 0.09234 |
| RELA | 681.127 | -0.942 | 0.351 | -2.680 | 0.00736 | 0.08594 |
| TBK1 | 362.809 | -0.939 | 0.351 | -2.679 | 0.00739 | 0.08594 |
| TOLLIP | 707.350 | -0.929 | 0.275 | -3.381 | 0.00072 | 0.02255 |
| MALT1 | 538.740 | -0.920 | 0.310 | -2.966 | 0.00302 | 0.05346 |
| IL4R | 1152.225 | -0.891 | 0.310 | -2.877 | 0.00402 | 0.06444 |
| TRAF4 | 861.409 | -0.857 | 0.334 | -2.565 | 0.01033 | 0.09868 |
| IFNGR1 | 2065.061 | -0.771 | 0.236 | -3.263 | 0.00110 | 0.03118 |
| CASP2 | 207.125 | 0.650 | 0.240 | 2.709 | 0.00676 | 0.08594 |

**Supplementary Table 3. Comparison of relative change in blood eosinophil counts, poly (I:C)-induced release of IL-33 by HBECs, and all variables included in the clustering analysis between hieralchical groups**

| Variables | high-inflammation (n=6) | low-inflammation (n=7) | p-value |
|---|---|---|---|
| Relative change in blood eosinophil counts after Tezepelumab ((after-before)/before) | -0.29 (0.57) | 0.07 (0.30) | **0.046** |
| IL33 (pg/mL)_PIC_Before Tezepelumab | 20.17 (4.55) | 13.21 (3.61) | **0.0140** |
| **Included in the cluster analysis** | | | |
| FeNO (ppb) before Tezepelumab | 48.83 (46.32) | 20.51 (17.44) | 0.1014 |
| TNFα (pg/mL)_PIC_Before Tezepelumab | 1116 (906) | 13.21 (3.60) | **0.0082** |
| IL4 (pg/mL)_PIC_Before Tezepelumab | 2.24 (0.52) | 1.23 (0.44) | **0.003** |
| IL13 (pg/mL)_PIC_Before Tezepelumab | 46.50 (4.40) | 33.86 (4.50) | **0.0003** |
| TSLP (pg/mL)_PIC_Before Tezepelumab | 26.05 (12.83) | 17.41 (7.80) | 0.1265 |

Data are presented as mean (SD) unless otherwise is expressed. Non-parametric Mann-whitney tests were performed.

**Supplementary Table 4. Clinical and Demographic characteristics of the stratified study population**

| | **High-inflammation** | **Low-inflammation** | **p-value** |
|---|---|---|---|
| N | 6 | 7 | |
| **Demographic characteristics** | | | |
| Age | 47.5 (21.01) | 42.86 (21.36) | 0.6562 |
| Sex (M/F) | 3/3 | 4/3 | 0.7821 |
| BMI (Kg/m$^2$) | 29.22 (3.16) | 26.54 (5.07) | 0.366 |
| **Lung function and asthma control** | | | |
| FEV1(%) | 97.5 (11.33) | 89.14 (9.99) | 0.1667 |
| FVC(%) | 111.70 (18.73) | 104.90 (13.67) | 0.6055 |
| FEV1/FVC(%) | 0.73 (0.10) | 0.70 (0.03) | 0.8089 |
| ACQ6 | 2.30 (1.00) | 2.20 (0.68) | 0.9138 |
| **T2 and inflammation markers** | | | |
| Blood eosinophil counts ($10^3$ cells/uL) | 0.41 (0.24) | 0.23 (0.14) | 0.1014 |
| FeNO (ppb) | 55.19 (48.76) | 20.51 (17.44) | 0.1014 |
| IgE (IU/mL) | 386.8 (497.8) | 114.1 (133.2) | 0.1512 |
| CRP | 3.50 (3.73) | 2.29 (1.80) | 0.9126 |

Data are presented as mean (SD) unless otherwise is expressed. Non-parametric Mann-whitney test was performed to compared continuos variables and Chi-square test to compare proportions
